# The Diagnostic Pitfalls of Invasive Breast Lobular Carcinomas Around Involuting Fibroadenomas With Coarse Calcifications: A Case Report

**DOI:** 10.7759/cureus.94088

**Published:** 2025-10-08

**Authors:** Taisuke Yagyu, Shoji Oura

**Affiliations:** 1 Department of Surgery, Kishiwada Tokushukai Hospital, Kishiwada, JPN

**Keywords:** breast cancer, coarse calcification, fibroadenoma, invasive lobular carcinoma, medio-lateral view mammography

## Abstract

A 69-year-old female with an involuting fibroadenoma (FA) was referred to our hospital for detailed examination of mammographic abnormalities around the coarse calcifications. Despite the lack of meaningful findings on medio-lateral oblique (MLO) view mammography, cranio-caudal (CC) view mammography showed spiculae around the calcifications. Ultrasound showed an oval mass with indistinct margins, disruption of the anterior borders of the mammary gland, and haloes just above the disruption areas. MRI depicted an irregular mass that showed low signals on T1-weighted images, slightly high signals on fat-suppressed T2-weighted images, and a plateau pattern on dynamic studies. Under the tentative diagnosis of breast cancer around the calcified FA, we performed a vacuum-assisted biopsy of the target lesion.

Pathological examination showed small atypical cells growing in linear and scattered fashions with connective tissue proliferation, leading to the diagnosis of invasive lobular carcinoma (ILC). The patient, therefore, underwent nipple sparing mastectomy and sentinel node biopsy followed by immediate breast reconstruction using the extended latissimus dorsi musculocutaneous flap. Postoperative pathological study revealed four ILC foci up to 18 mm in size around the intracanalicular type FA with massive calcifications. Immunostaining showed estrogen and progesterone receptor positivity (both Allred score 8), human epidermal growth factor receptor type 2 negativity, and a Ki-67 labelling index of 5%. The patient recovered uneventfully, was discharged on the 14th day after the operation, and is scheduled for long-term outpatient follow-up on endocrine therapy. Diagnostic physicians should note that ILCs can develop around the calcified FAs and are prone to being overlooked on MLO view mammography.

## Introduction

Invasive lobular carcinomas (ILCs) naturally develop from mammary lobules. They can be multicentric and bilateral, and characteristically metastasize to rare organs and sites such as the gastrointestinal tract and retroperitoneum [1,2]. ILCs generally have abundant fibrous components, which can cause distortion of the mammary gland and give ILCs a hardness. Scirrhous-type invasive ductal carcinomas also have a large amount of fibrous components, often leading to challenges in terms of differential diagnosis between scirrhous carcinomas and ILCs, not only pathologically but also radiologically. Scirrhous-type invasive ductal carcinomas, however, less often have multicentricity and bilaterality, and rarely develop distant metastasis to uncommon organs and tissues.

Fibroadenomas (FA) have both epithelial and connective tissue components and are the most common benign breast tumors. Despite the known frequent harboring of MED12 mutations [3], i.e., a gene encoding part of the RNA polymerase II mediator complex found in more than half of uterine myomas, the exact pathogenesis of FA remains unknown. Fibroadenomas grow in an estrogen-dependent manner and, therefore, do not grow larger after menopause, and frequently develop coarse calcifications [4]. Similar to ILCs, fibroadenomas develop from mammary lobules. Nevertheless, it is rare for breast cancer to develop within fibroadenomas, and when it does, it is most often ductal carcinoma in situ [5]. No studies, however, have reported a lobular carcinoma encompassing a calcified FA to date. We report a case of ILC surrounding a calcified fibroadenoma, which had obscured the ILC.

## Case presentation

**Figure 1:**
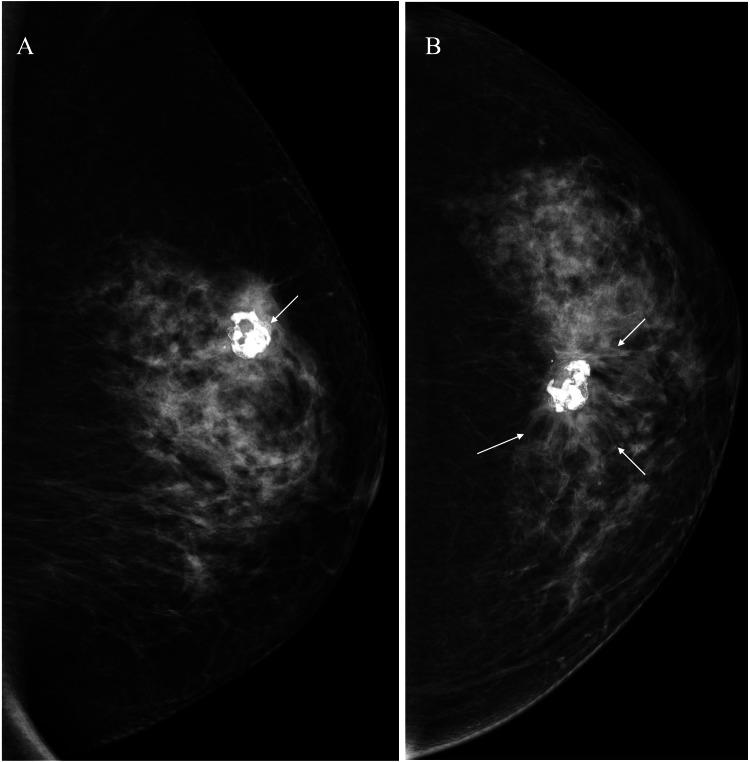
Mammography findings (A) Medio-lateral oblique view mammography only showed coarse calcifications (arrow). (B) Cranio-caudal view mammography showed evident distortion (arrows) around the calcifications

A 69-year-old female with no particular medical history, whose mother had a history of pancreatic cancer, had been aware of a benign tumor in her left breast for more than 20 years. She was referred to our hospital due to the abnormal mammography findings around the benign tumor. Mammography showed a dense breast pattern and no abnormalities except for the coarse calcifications on the medio-lateral oblique (MLO) view. Cranio-caudal (CC) view mammography, however, showed spiculae around the calcifications (Figure 1).

**Figure 2:**
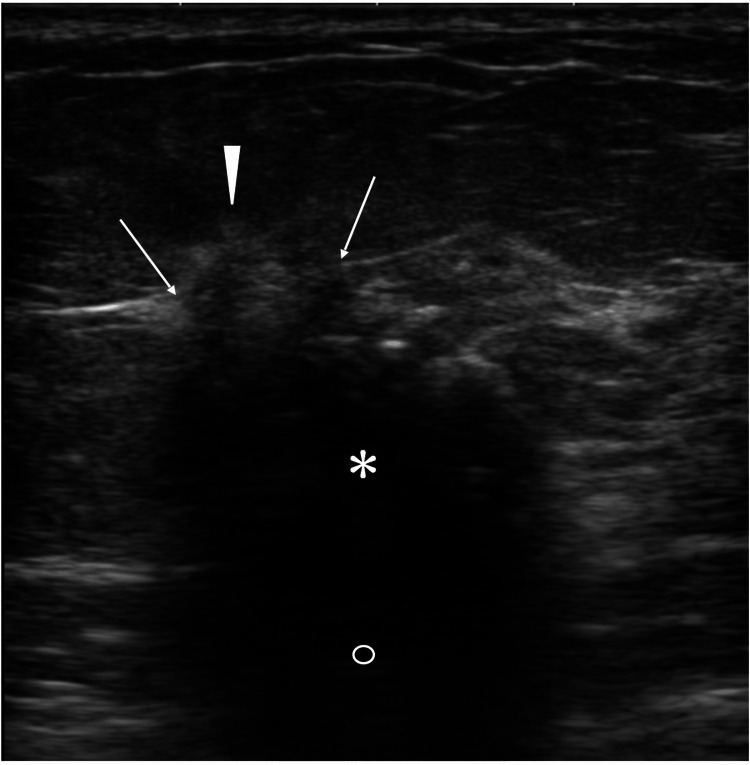
Ultrasound findings Ultrasound showed an oval mass (asterisk) with indistinct margins, internal low echoes, attenuated posterior echoes (open circle), disruption of mammary gland anterior borders (arrows), and focal haloes (arrowhead)

Ultrasound showed an oval mass, 18 mm in size, with indistinct margins, disruption of the anterior borders of the mammary gland, and haloes just above the disruption areas (Figure 2).

**Figure 3:**
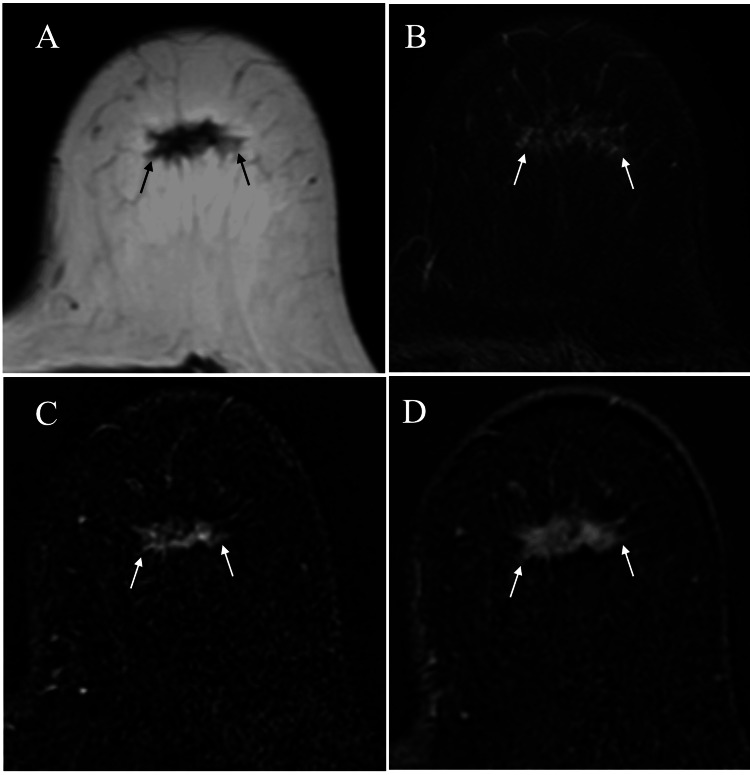
MRI findings MRI of the mass showed low signals (arrows) on T1-weighted images (A), faint high signals (arrows) on T2-weighted images (B), and early (C) and retained (D) enhancement (arrows) on dynamic studies MRI: magnetic resonance imaging

MRI depicted an irregular mass that showed low signals on T1-weighted images, slightly high signals on fat-suppressed T2-weighted images, and a plateau pattern on dynamic studies (Figure 3).

**Figure 4:**
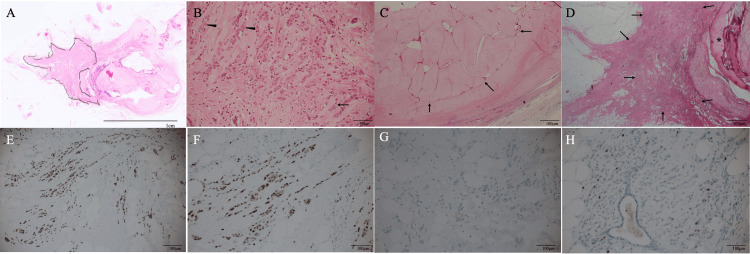
Pathological findings (A) Low magnified view showed invasive lobular carcinoma cells (closed areas) and the involuting fibroadenoma (asterisk) (H.E. ×4). (B) Magnified view showed atypical cells growing in linear (arrow) and cord-like (arrowheads) fashions against the abundant fibrous backgrounds (H.E. ×200). (C) Fibroadenoma (arrows) was of an intracanalicular phenotype (H.E. ×40). (D) Atypical cells (arrows) surrounded the coarse calcifications (asterisk) (H.E. ×40). (E) Immunostaining showed that invasive lobular carcinoma cells (brownish cells) had high estrogen receptor positivity (×200). (F) Immunostaining showed that invasive lobular carcinoma cells (brownish cells) had high progesterone receptor positivity (×200). (G) Tumor cells showed no positivity of human epidermal growth factor receptor on immunostaining (×200). (H) Tumor cells had a low Ki-67 labelling index of 5% (×200)

Under the tentative diagnosis of breast cancer around the calcified FA, we performed a vacuum-assisted biopsy of the target lesion. Pathological examination showed small atypical cells growing in linear and scattered fashions with connective tissue proliferation, leading to the diagnosis of ILC. Immunostaining showed that the ILC was a luminal subtype breast cancer with a low proliferating index. Based on the patient's wishes, we operate on the patient with nipple sparing mastectomy and sentinel node biopsy, followed by immediate breast reconstruction using the extended latissimus dorsi musculocutaneous flap. Postoperative pathological study revealed four ILC foci up to 18 mm in size and an intracanalicular type FA with massive calcifications. Immunostaining showed estrogen and progesterone receptor positivity (both with an Allred score of 8), human epidermal growth factor receptor type 2 negativity, and a low Ki-67 labelling index of 5% (Figure 4).

The patient recovered uneventfully; she was discharged on the 14th day after the operation and is scheduled for a 10-year outpatient follow-up on endocrine therapy.

## Discussion

Coarse calcifications observed in this case could be either dystrophic calcifications or those developed in postmenopausal involuting fibroadenomas. Dystrophic calcifications can develop through various mechanisms, such as surgery to the breast and degenerative changes of the mammary gland. Especially, breast surgery can cause spiculae around the operative site, which often mimic breast cancer-induced spiculae. Coarse calcifications after breast surgery, however, are generally accompanied by evident surgery-related ultrasound findings and, therefore, were easily negated in this case. Coarse calcifications are naturally composed of calcium. Large calcifications have such high acoustic impedance that they reflect almost all ultrasound waves, preventing the diagnostic physicians from properly evaluating the tissue under the large calcifications on ultrasound. On the other hand, calcifications have a high X-ray attenuation coefficient on mammography, showing them as white and making small lesions in front of or behind them, and are often overlooked, like in this case.

Unlike pleomorphic ILCs [6], classic ILCs generally have better clinical outcomes than invasive ductal carcinomas [7]. In fact, the Ki67 labelling index was very low at 5% in this case. Due to the lack of detailed information about our patient's past screening mammography, we could not evaluate how coarse calcifications had obscured this less aggressive ILC. However, it cannot be denied that the Japanese standard screening mammography method, i.e., MLO view mammography without CC view mammography for patients aged 50 years or older [8], might have led to the underdetection of ILC in this case.

It is well known that MLO view mammography shows the breast in a more expanded form than CC view mammography. Many diagnostic physicians, however, have empirically known that the ILCs are more clearly visualized on CC view mammography than on MLO view mammography. Unfortunately, no research has proved to date why this phenomenon occurs. Clear tumor margins may be obscured by overlapping with the mammary gland and can often become evident by increased breast compression.

We have already clarified that fibrous components, when present at the mass borders mixed with cancer cells, can obscure tumor margins [9,10]. ILCs, therefore, have highly indistinct margins, which never become distinct with more breast compression. Conversely, when tumor margins show spicula-like structures, they become more unclear on the MLO view mammography due to the further magnification of unclear margins. In addition, the breasts are thinner on the MLO view mammography than on the CC view mammography, and make the mass opacity further lower, leading to more difficult identification of ILCs on MLO view mammography. These are presumably the main reasons why ILCs are less detectable on MLO view mammography.

Simple fibroadenomas are not known to increase the risk of breast cancer [11]. The clinical significance of why this ILC developed just around the calcified fibroadenoma is unclear. However, it is imperative that screening mammography with MLO and CC views be performed even for elderly people to avoid underdetection of ILCs around the coarse calcifications, if present.

## Conclusions

Classic-type ILCs surrounding calcified fibroadenomas are extremely rare and can be overlooked on screening mammography. Therefore, if known calcified fibroadenomas are present, screening mammography should be performed by using both MLO and CC views, even for elderly people. In addition, diagnostic physicians should note that ILCs can be detected more easily on CC view mammography than MLO view mammography.
